# Is There a Role for Daratumumab Retreatment in Patients with Relapsed/Refractory Multiple Myeloma?

**DOI:** 10.3390/biomedicines13010207

**Published:** 2025-01-15

**Authors:** Davor Galusic, Ivan Krecak, Viktor Blaslov, Andela Krstulovic Opara, Toni Valkovic, Sandra Basic Kinda

**Affiliations:** 1 Division of Hematology, University Hospital of Split, 21000 Split, Croatia; davorgalusic@net.hr (D.G.); viktor.blaslov@gmail.com (V.B.); andjela.krop@gmail.com (A.K.O.); 2 School of Medicine, University of Split, 21000 Split, Croatia; 3 Department of Internal Medicine, General Hospital of Sibenik, 22000 Sibenik, Croatia; 4 Faculty of Medicine, University of Rijeka, 51000 Rijeka, Croatia; toni.valkovic@medri.uniri.hr; 5 University of Applied Sciences Sibenik, 22000 Sibenik, Croatia; 6 Department of Internal Medicine, Pula General Hospital, 52100 Pula, Croatia; 7 Division of Hematology, University Hospital Centre Zagreb, Kispaticeva 12, 10000 Zagreb, Croatia

**Keywords:** multiple myeloma, relapse, daratumumab, retreatment

## Abstract

Multiple myeloma (MM) is a hematologic disease characterized by the clonal expansion of malignant plasma cells that accumulate in the bone marrow, leading to osteolytic bone disease, hypercalcemia, anemia, and renal dysfunction. Daratumumab was the first monoclonal anti-CD38 antibody approved for the treatment of MM, initially in relapse/refractory settings and, more recently, for newly diagnosed patients. Increased first-line usage of daratumumab will also substantially change treatment approaches for patients with relapsed/refractory disease. Due to the cost and availability of bispecific T cell redirecting antibodies (BsAbs) and chimeric antigen receptor T cell therapy (CAR-T) in real-life settings in many countries, retreatment with daratumumab in subsequent lines of therapy might be a reasonable choice. Data regarding efficacy and optimal combinations of daratumumab retreatment are lacking, and here we provide a short literature review of available data. We identified only a small number of articles based on retrospective analysis of medical records in real-life settings. A strong consistency in results regarding response rates and treatment duration was noticed among mainly heavily pre-treated MM patients, with approximately half of patients achieving at least partial remission (PR) after retreatment with daratumumab-based protocol. The duration of treatment and time to the next treatment for retreatment episodes were considerable and consistent with clinical expectations for later lines of therapy. The analysis of data in this literature review indicates that daratumumab retreatment may provide meaningful clinical benefit to some patients with relapsed/refractory MM despite having prior exposure. However, further research is needed to identify clinical and biological parameters that may predict favorable responses to daratumumab retreatment.

## 1. Introduction

Multiple myeloma is a hematologic disease characterized by the clonal expansion of malignant plasma cells that accumulate in the bone marrow leading to osteolytic bone disease, hypercalcemia, anemia, and renal dysfunction [1]. Some patients with high tumor burden are presented with life-threatening complications such as hyperviscosity syndrome which is usually caused by increased circulating serum immunoglobulins leading to neurologic symptoms, bleeding, or thromboembolic incidents [2]. The use of proteasome inhibitors (PI) in combination with immunomodulatory drugs (IMiD) brought a significant improvement in patient outcomes, especially in patients eligible for autologous stem cell transplantation [3,4]. Daratumumab is a human IgGκ that targets CD38 and has a direct antitumor and immunomodulatory activity [5,6,7,8]. It was the first monoclonal antibody approved for the treatment of multiple myeloma, initially as monotherapy for heavily pre-treated patients based on GEN501 and SIRIUS trials [9,10]. Subsequently, the addition of daratumumab to standard protocols demonstrated significant clinical benefit in several phase 3 clinical trials involving patients with relapsed/refractory disease (CASTOR and POLLUX trials) and newly diagnosed myeloma patients (ALCYONE and MAIA trials) [11,12,13,14]. More recent trials emphasized the role of daratumumab in quadruplet combinations for newly diagnosed transplant-eligible patients. In the CASSIOPEIA trial, the addition of daratumumab to bortezomib, thalidomide, and dexamethasone (D-VTd) has shown clinical benefit in comparison to bortezomib, thalidomide, and dexamethasone (VTd) alone [15]. In the phase 2 GRIFFIN trial quadruplet with daratumumab, bortezomib, lenalidomide, and dexamethasone (D-VRd) improved the depth of response and progression-free survival (PFS) in comparison to bortezomib, lenalidomide, and dexamethasone (VRd). The efficacy of D-VRd has also been recently confirmed in PERSEUS phase 3 clinical trial [16,17]. These results have led to daratumumab approval for frontline treatment in newly diagnosed patients, regardless of transplant eligibility [18]. Increased first-line (1L) usage of daratumumab is already seen in many countries and is surely expected to be more pronounced in the near future [19,20,21,22]. New treatment standards in front-line settings will also substantially change the treatment approaches for patients with relapsed/refractory disease. Bispecific T cell redirecting antibodies (BsAbs) and chimeric antigen receptor T cell therapy (CAR-T) are treatment options whose efficacy and safety profiles have already been established in many clinical trials in patients with relapsed or refractory disease [23,24,25,26,27]. However, the cost and availability of these therapies in real-life settings might be an important factor in many countries.

The CD38 molecule was the first target on tumor cells that was widely used in the treatment of newly diagnosed and relapsed/refractory disease thanks to the anti-CD38 monoclonal antibodies daratumumab and isatuximab [28,29,30,31]. The evolution of drug development based on CD38 targeting continues. So far, there is more preclinical evidence of the efficacy of anti-CD38-modified T cells, while some clinical studies investigating the efficacy and safety of anti-CD38 CAR–T cells in relapsed/refractory MM are in progress [32,33,34]. Also, there are very encouraging preclinical and clinical data on the efficacy and safety of bispecific CAR-T cell therapy targeting BCMA and CD38 [35]. Unfortunately, all of these drugs are in development, and immunotherapy that is currently approved is still unavailable to many patients worldwide due to cost and production limitations, which all speaks in favor of the practical importance of retreatment with anti-CD38 antibodies in real life. Immunotherapy retreatment in hematologic malignancies has already been shown to be effective, dominant in the treatment of relapsed and refractory lymphoproliferative disorders. Anti-CD20 and anti-CD30 antibodies are widely used as the retreatment of non-Hodgkin and Hodgkin lymphoma, respectively, as long as the tumor cells maintain targeted CD expression [36,37,38]. At the relapse, tumor plasma cells maintain CD38 expression, and retreatment with daratumumab could be the logical choice, especially if other treatment options are unavailable. It is also known that CD38 expression on tumor plasma cells recovers soon after the stopping treatment with anti-CD38 antibodies [39].

Different mechanisms of resistance to daratumumab have been described. One of the most frequently mentioned is the reduction of CD38 expression on myeloma cells and the clonal selection of CD38^dim^ tumor cells caused by treatment with anti-CD38 antibodies [40]. However, low CD38 levels on myeloma cells is a transient effect and it has already been shown that in 3 to 6 months after stopping treatment with anti-CD38 antibodies, CD38 expression on tumor plasma cells recovers [39]. Unfortunately, it is still not entirely clear how clinically relevant this effect can be [39]. Furthermore, the use of daratumumab can lead to the depletion of effector memory T cells and tumor-associated macrophages-type 1 as well as the downregulation of co-stimulatory CD28 expression on T cells, which altogether leads to further immunosuppression and worse control of tumor cells [40].

Because daratumumab combinations are approved in different combinations in the relapsed/refractory setting, retreatment with daratumumab in subsequent lines of therapy might be a reasonable choice.

Data regarding the efficacy and optimal combinations of daratumumab retreatment are lacking. Here we provide a short literature review of available data regarding daratumumab retreatment.

## 2. Materials and Methods

**Figure 1:**
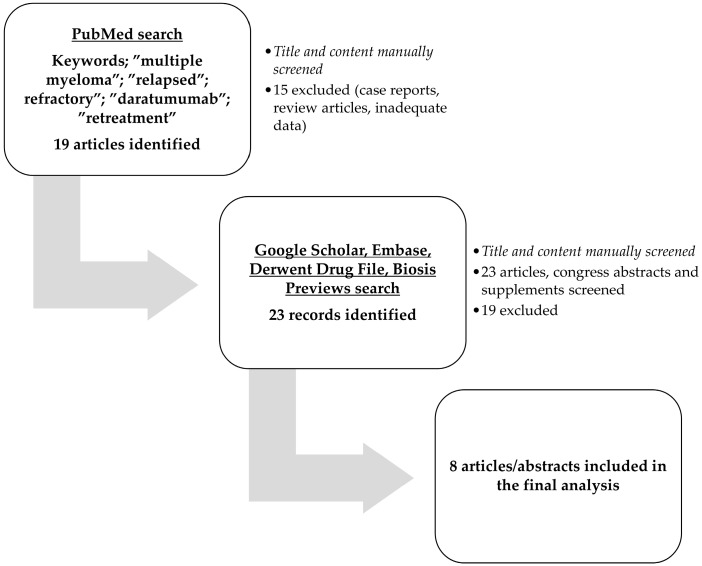
The study flowchart.

For this systematic review eligible articles were searched in PubMed using the keywords: “multiple myeloma” AND “relapsed” OR “refractory” AND “daratumumab” AND “retreatment” OR “re-treated” OR “retreated” OR “re-treatment”. Other databases searched were Google Scholar, Embase, Biosis Previews, and Derwent Drug File. All articles were manually checked for content, and eight articles were ultimately included in this review. All articles were published in English, and conference reports were not discarded. The literature search was conducted in June 2024. Article selection followed the recommendations of the Preferred Reporting Items for Systematic Reviews and Meta-analyses (PRISMA) (Supplementary Materials File S1). The study flowchart is illustrated in Figure 1.

## 3. Rationale for Retreatment in Multiple Myeloma and Literature Review

The data for retreatment with available drugs for myeloma are sparse. The most robust data are available for successful retreatment with bortezomib and lenalidomide [41,42]. Due to mostly retrospective data on retreatment, it is possible that the patients who had initially responded well to a given drug were preferentially chosen for retreatment. In particular, response rates at retreatment may be highest in patients who achieved a complete response (CR) to the initial bortezomib and in whom the first response was of relatively long duration [41,43,44,45]. It is also hypothesized that the efficacy of the retreatment with immunomodulatory drug (IMiD) may be due to immune-enhancing effects and not by direct antitumor effect [46]. Data regarding the efficacy of daratumumab retreatment are lacking. In the randomized clinical trial POLLUX, 11 patients were reported to receive a daratumumab-based regimen as the first subsequent therapy after treatment with daratumumab + lenalidomide + dexamethasone (DRd) [12,30]. However, data regarding outcomes of this particular subgroup of patients were not reported. The purpose of the phase 2 study NCT03871829 was to determine the efficacy of daratumumab in combination with carfilzomib and dexamethasone (DKd) in patients with relapsed/refractory multiple myeloma who had previously been exposed to daratumumab to evaluate daratumumab retreatment [47]. The study was discontinued as the data review committee recommendation was the early stop of the study for futility [48]. It is also unlikely to expect another randomized trial with daratumumab retreatment nowadays since there are a lot of other competitive trials with novel agents. Therefore, conducting results from a real-life setting is an important tool to evaluate the potential benefit of daratumumab retreatment in patients with relapsed/refractory MM.

Abdallah et al. (2023) conducted a single-center retrospective database review to analyze the efficacy and safety of retreatment with daratumumab in patients with relapsed/refractory multiple myeloma (RRMM) who were refractory to daratumumab, and compared the response data after retreatment with the response data after the first daratumumab-based line of therapy. Overall, 43 patients were included. Patients had received a median of two lines of therapy (range, 1–8) before their first daratumumab-based regimen and a median of four lines of therapy (range, 2–14) before the daratumumab-based retreatment. All daratumumab-retreated patients received combination therapy with either pomalidomide, carfilzomib, bortezomib, or lenalidomide. The median duration of follow-up after daratumumab IV retreatment was 19.5 months. In the retreatment group, the overall response rate (ORR), median progression-free (PFS), and overall survival (OS) were 49%, 7.97, and 32.6 months, respectively. The median OS and PFS were NR among patients who achieved at least partial remission (PR). In daratumumab, the naïve group ORR was 65% and the median time to relapse or progressive disease (PD) was 6.77 months [49]. The main limitation of this study is a retrospective design, which relies on a database review of a small patient cohort.

Girvan et al. (2022) conducted a retrospective study based on health records from United States community-based oncology centers. A total of 97 patients who received a daratumumab-based regimen and subsequent retreatment with daratumumab were included. The primary objective was to determine the proportion of patients treated and retreated with daratumumab overall and by line of therapy (LOT). The secondary objectives were duration of treatment (DOT) and time to next treatment (TTNT). The most common daratumumab regimen used for retreatment was a combination with IMiD (43%), followed by daratumumab + PI + IMiD (33%) and daratumumab + PI (15%). Among retreated patients, the median DOT was 254 days and 196 days for the first daratumumab treatment and retreatment period, respectively. The median DOT by LOT for the first daratumumab treatment period ranged from 173 days (the fourth line and beyond [4L+]) to 288 days (1L). The median DOT by LOT for the daratumumab retreatment period ranged from 149 days (4L+) to 304 days (1L). The median TTNT was 308 days and 457 days for the first daratumumab treatment and retreatment period, respectively. The median TTNT by LOT for the first daratumumab treatment period ranged from 286 days (4L+) to 434 days (1L). The median TTNT by LOT for the daratumumab retreatment period ranged from 256 days (3L) to not yet reached (1L) [50]. This study included a significant number of patients from centers where clinical trials are usually not conducted. Furthermore, these patients had not undergone autologous stem cell transplantation, which makes them representative of relapsed/refractory patients in a real-life setting. It would be interesting if the time period between initial and subsequent daratumumab treatment were reported.

Ciardiello et al. (2022) presented (at the American Society of Clinical Oncology [ASCO] Annual Meeting) the results of a single-center retrospective chart review comparing the efficacy of second-line treatment with daratumumab-based regimens in patients who had previously received induction treatment with daratumumab-based (cohort 1) vs. daratumumab-free induction (cohort 2). Overall, 33 patients were included. Among six patients in cohort 1, five received daratumumab induction in combination with carfilzomib, lenalidomide, and dexamethasone (D-KRd), and one patient daratumumab with lenalidomide and dexamethasone (DRd). The median duration between the last dose of daratumumab in the 1L of therapy and the first dose of daratumumab in the 2L of therapy was 17.5 months. All patients in cohort 2 (N = 27) received induction with carfilzomib, lenalidomide, and dexamethasone (KRd). The most common regimen used in 2L was a combination of daratumumab with pomalidomide and dexamethasone (DPd) in 18 (55%) patients. ORR rates were similar among groups, 83% and 78% for cohort 1 and cohort 2, respectively [51]. This retrospective chart included only six patients who were retreated with daratumumab and the results were only reported as a conference paper. Response rates were quite favorable, which could be associated with a longer period between the initial and the second daratumumab treatment line.

Szabo et al. (2022) conducted a retrospective real-world study that evaluated the life expectancy and clinical outcomes in patients who discontinued their first daratumumab-based regimen. They identified 474 patients who discontinued the daratumumab-based regimen. The most common initial daratumumab-based regimen was DRd in 44%, followed by daratumumab monotherapy in 32%, daratumumab + bortezomib + dexametason (DVd) in 13%, and other daratumumab-based regimens in 11% of patients. The most common reasons for discontinuation were PD (42%), toxicity (11%), and insufficient response (8%). Among 375 patients who received a subsequent line of therapy, 192 (51%) were retreated with a daratumumab-based regimen. The most frequently used regimens in patients retreated with daratumumab were DPd (30%), DRd (23%), and DVd (12%). The median TNT was 4.6 months in both groups. ORR in patients who received retreatment with daratumumab IV and in those who did not was 48% and 41%, respectively. Retreatment with daratumumab was associated with longer OS (median OS 23.6 vs. 11.3 months for patients with daratumumab retreatment and without daratumumab retreatment respectively, *p* < 0.0001) [52]. Even though a significant number of patients were analyzed in this study, more than 40% of retreated patients were initially treated with daratumumab as a monotherapy which makes this cohort less representative in nowadays real-life settings. Furthermore, in more than half of the patients in this cohort, initial daratumumab treatment was not stopped due to refractoriness, therefore better outcomes after retreatment should be expected.

Atrash et al. (2021) conducted a multicenter retrospective chart review to evaluate the clinical outcomes of patients with multiple myeloma who received daratumumab-based regimens across different lines of therapy. Overall, 299 patients were included, of whom 19 were retreated with daratumumab (resumed a daratumumab-based regimen after an interruption of ≥90 days). The mean duration of daratumumab interruption was 258 days (range, 93–644). During the interruption of daratumumab, 14 [73.7%] patients received non-daratumumab-based regimens. Among retreated patients, all but one patient had their first daratumumab-based treatment segment in the third line (3L) of therapy or later and the most common regimen used for the subsequent daratumumab-based treatment segment was DPd (26.3%). Six patients (31.6%) remained on the same daratumumab-based regimen before and after interruption. Among these, four patients did not receive any treatment during the gap. The mean duration of the initial segment was 195 days and the mean duration of the second segment was 103 days. ORR rates of the retreatment cohort were 66.7% and 52.9% for the first and second segments of daratumumab-based regimen, respectively [53]. The main limitation of this study, among retrospective design, is the small number of patients included. This cohort is also heavily pre-treated but the duration of daratumumab interruption was more than 8 months.

Nooka et al. (2019) conducted a retrospective safety and efficacy analysis of the DPd regimen. The analysis also presented long-term follow-up results of patients that were either refractory to pomalidomide or daratumumab or both; or without prior exposure to daratumumab and pomalidomide. Among the subgroup of 12 patients who were daratumumab and pomalidomide refractory, 4 of them responded when they were retreated with DPd combination [54]. These results are also very limited due to small sample size.

Kim et al. (2019) presented (at the International Myeloma Workshop [IMW]) the results of a retrospective chart review evaluating the efficacy and safety of re-initiation of daratumumab after an interruption in previous daratumumab-based treatment. They identified 19 patients who were retreated with the daratumumab-based regimen. Among the included patients, treatment was interrupted due to PD in 8 (42%) patients and due to delay in the same therapy in 11 (58%) patients. Patients who had treatment interruption due to PD had received other therapies before restarting daratumumab. The duration of therapy upon re-initiation was 1 to 14 doses. In this heavily pre-treated population, the response rates were variable, but generally low [55]. This retrospective chart included a small number of patients who were retreated with daratumumab and the results were only reported as a conference paper.

Souren et al. (2024) conducted a single-center retrospective study in which they identified a total of 293 patients treated with daratumumab-based regimens. Among them, 23 (8%) were retreated with daratumumab, mostly as the 4th or subsequent treatment line. The median durations of the first and subsequent daratumumab treatment were similarly long. Daratumumab retreatment was effective, with responses declining only gradually from its first use to subsequent first and second retreatment with 64%, 46%, and 43%, respectively. Interestingly, comparable progression-free survival rates were observed at 11.5, 12 months, and not reached, respectively. Consistently, adverse events per daratumumab line did not increase [56]. Initial daratumumab was dominantly combined with PI which could be one of the reasons for the relatively shorter median PFS in comparison to combinations with IMiD in subsequent treatment lines. The duration between daratumumab-based regimens were about 8 months and most of the patients were therapy-free in that period. It would be interesting if the reasons for daratumumab discontinuation were also reported.

A brief overview of selected studies that focused on daratumumab retreatment in patients with relapsed/refractory multiple myeloma is summarized in Table 1.

## 4. Discussion

To the best of our knowledge, our short literature review is the first to systemically collect data regarding daratumumab retreatment among patients with relapsed/refractory multiple myeloma after previous relapse or refractoriness with daratumumab or after a treatment-free interval. No published prospective or randomized trials are available, and we identified only a small number of articles based on retrospective single- or multicenter analyses of medical records in real-life settings. Even though a number of patients with data regarding the efficacy of daratumumab retreatment analyzed in these studies is rather small, we noticed a strong consistency in results regarding ORR and treatment duration. Approximately half of the patients (21 of 43) responded to a daratumumab-based retreatment protocol based on a single-center retrospective chart review conducted by Abdallah and colleagues [49]. Almost identical results were observed in a small cohort of 19 retreated patients identified in the multicenter analysis of patients who received daratumumab-based regimens, as well as in the analysis of a much larger cohort (192 retreated patients), which evaluates clinical outcomes in patients who discontinued daratumumab-based regimen [52,53]. The median OS reported in two studies ranged between 24 and 33 months [49,52]. When we consider the fact that this population is mainly heavily pre-treated, these response rates are even more pronounced. In only one single-center experience, early retreatment with daratumumab was identified in six patients. It is logically expected that response rates evaluated in early retreatment would be higher than in heavily pre-treated patients, but in this study, it was also noticed that response rates on daratumumab-based regimens in the second line are comparable in patients who underwent daratumumab induction and those who did not [51]. The most common regimen used in daratumumab retreatment was DPd. This protocol seems to be associated with better outcomes with up to one-third of relapsed/refractory patients who responded even though they had been previously refractory to both daratumumab and pomalidomide [54]. The other important outcome markers evaluated in these studies are DOT and TTNT. In retrospective analysis conducted by Girvan and colleagues DOT and TTNT for daratumumab retreatment period were considerable and consistent with clinical expectations for later lines of therapy, suggesting that patients continue to receive substantial clinical benefit with daratumumab despite prior exposure [50]. These results are promising and daratumumab retreatment could be a reasonable option for some relapse refractory multiple myeloma patients, especially if new treatment options like BsAbs or CAR-T are not available. Earlier lines of daratumumab retreatment, previous favorable responses to daratumumab, and new combinations with PI and IMiD are expected to be associated with better outcomes. Furthermore, it would be interesting to periodically access CD38 expression in patients with relapsed/refractory MM and daratumumab retreatment would have more sense if the CD38 expression is pronounced and if a longer time had passed since the previous daratumumab usage. The combination with PI or IMiD that was not previously used should be the treatment of choice whenever possible. In the future, new daratumumab combinations could also be available, like the combination with BsAbs talquetamab, of which efficacy is currently being evaluated in patients with relapsed/refractory MM.

Daratumumab has a range of adverse effects, some of which are not commonly associated with other combination chemotherapy. It has been associated with a high rate of infusion-related reactions (IRR). About half of the patients treated with daratumumab experienced a reaction, mostly during the first infusion, though infusion reactions may also occur with later infusions [57]. Some of the most commonly reported side effects of daratumumab were also myelosuppression, atrial fibrillation, peripheral neuropathy, fatigue, peripheral edema, allergic rhinitis, nasopharyngitis, pyrexia, dyspnea, pneumonia, GI disorders (nausea, constipation, diarrhea), headache, and hypertension [57].

Since there are very few studies on this topic, it is not possible to compare and categorize patients according to their clinical features. Additional studies with a larger number of patients are needed as there are many confounding factors in addition to missing key comparison features (such as performance status, age, comorbidities, staging, high genetic risk, disease biology, response to prior therapy, etc.). However, at this time it is only possible to analyze rough data on the effectiveness of daratumumab retreatment, but even these rough data may be useful to the clinician when there are no other available treatment options. Furthermore, it is unlikely to expect randomized trials in which the effectiveness of daratumumab retreatment would be properly evaluated and distinguished from the effect of additional drugs.

The main limitation of this review is the limited number of articles with generally small cohorts of daratumumab retreated patients, and all conclusions are based on retrospective analysis of medical records where the intrinsic risk of bias exists. All of these studies included a very heterogeneous population of relapsed/refractory MM patients. Some patients were heavily pre-treated (even more than 10 lines of therapy), while others received daratumumab retreatment as the second treatment line. Additionally, daratumumab was most frequently used in combination with other compounds and both prior and subsequent treatment regimens were quite heterogeneous among different studies, making it difficult to comprehend whether treatment responses were related specifically to re-exposure to daratumumab or possibly to other agents and depth of response to prior regimens as well. Performance status, age, and comorbidities were also likely important factors in daratumumab retreatment consideration in real-life practice, together with daratumumab availability and insurance restrictions. Nevertheless, we believe that this short review could be helpful to hematologists in making clinical decisions for the treatment of relapsed/refractory patients in everyday practice.

## 5. Conclusions

In conclusion, retreatment with daratumumab in combination with different classes of anti-myeloma drugs may benefit a proportion of relapsed/refractory MM patients. The analysis of data in this short literature review indicates that some patients with relapsed/refractory MM may have clinical benefits with daratumumab retreatment despite prior exposure. Further research is needed to identify clinical and biological predictors of favorable treatment responses to daratumumab retreatment in this clinical setting.

## Figures and Tables

**Table 1:** An overview of selected studies on daratumumab retreatment in relapsed/refractory multiple myeloma patients.

Authors	Study Design	No of pts	Prior Treatment	Daratumumab Retreatment Regimen	Key Study Findings	PFS	OS
Abdallah et al. [49]	Single-center retrospective study	43	Median 4L (range 2–14L)	Dara-Pd (44%) Dara-Kd (40%) Dara-Rd (44%) Dara-Vd (2%)	OR 49%	7.97 months	32.6 months
Girvan et al. [50]	Multi-center retrospective study	97	21 (22%) 1L 38 (39%) 2L 23 (24%) 3L 15 (15%) 4L+	Dara-IMiD (43%) Dara-IMiD-PI (33%) Dara-PI (15%)	DOT 196 days TTNT 457 days	N/A	N/A
Ciardiello et al. [51]	Single-center retrospective study	6	1	Dara-KRd = 5 Dara-Rd = 1	OR 83%	Not reached	N/A
Szabo et al. [52]	Multi-center retrospective study	192	Dara-Rd (43.5%) Dara-mono (32.1%) Dara-Vd (13.3%) Dara-other (11.2%)	Dara-Pd (30%), Dara-Rd (23%) Dara-Vd (12%).	TNT 4.6 months OR 48%	N/A	23.6 months
Atrash et al. [53]	Multi-center retrospective study	19	3L+	Dara ± d (31.6%) D-Pd (26.3%)	DOT 103 days OR 52.9%	N/A	N/A
Nooka et al. [54]	Single-center retrospective study	34	Group 1 (Dara- and-or POM refractory (n = 22) Group 2 (Dara- and POM refractory, n = 12)	Dara-Pd	Group 1 OR 40.9% Group 2 OR 33.3%	5.2 months for group 1 3.3 months for group 2	15.2 months for group 1 13 months for group 2
Souren et al. [56]	Single-center retrospective study	23	4L+ (91%)	Dara (4%) Dara + PI (49%) Dara + IMID (19%) Dara + other (28%)	ORR 46% and 43% for the 1st and the 2nd daratumumab retreatment, respectively	12 months and not yet reached for the 1st and the 2nd daratumumab retreatment, respectively	N/A

PFS = progression-free survival, OS = overall survival, DOT = duration of treatment, TTNT = time to next treatment, OR = overall response, Dara = daratumumab, Pom = pomalidomide, V = bortezomib, K = carfilzomib, IMID = immunomodulatory drug, PI = proteasome inhibitor, d = dexamethasone, N/A = not available.

## Supplementary Materials

The following supporting information can be downloaded at: https://www.mdpi.com/article/10.3390/biomedicines13010207/s1.
