# Supplementary figures and images for: Is There a Role for Daratumumab Retreatment in Patients with Relapsed/Refractory Multiple Myeloma?
Source: Biomedicines. 2025 Jan 15;13(1):207. doi: 10.3390/biomedicines13010207 (PMC11762825; doi:10.3390/biomedicines13010207)

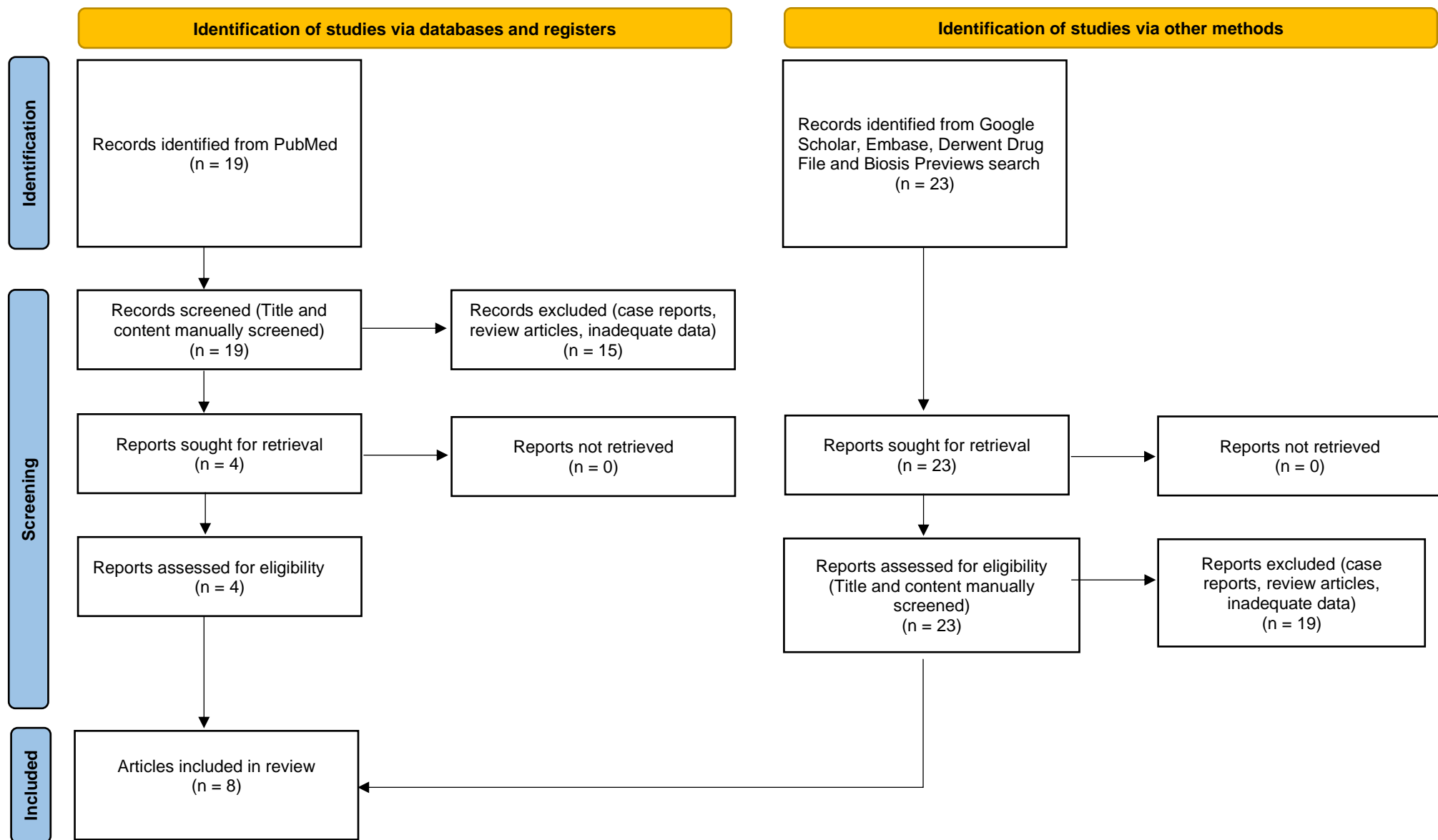

Supplement: Supplementary file 1 [file biomedicines-13-00207-s001.zip › biomedicines-3417546-supplementary.pdf]
